# Simulated dust activity in typical time periods of the past 250 million years

**DOI:** 10.1016/j.fmre.2024.02.004

**Published:** 2024-02-13

**Authors:** Qifan Lin, Yonggang Liu, Jiaqi Guo, Xiang Li, Jiawenjing Lan, Haoyue Zuo, Ming Zhang, Jian Zhang, Zhouqiao Zhao, Shuai Yuan, Xiujuan Bao, Yongyun Hu

**Affiliations:** Laboratory for Climate and Ocean-Atmosphere Studies, Department of Atmospheric and Oceanic Sciences, School of Physics, Peking University, Beijing 100871, China

**Keywords:** Dust, Pangea, Climate, Land plant evolution, Mesozoic, Triassic, Cretaceous

## Abstract

The dust cycle in three typical time periods of the past 250 Myr, namely 240 Ma, 130 Ma and 80 Ma, is studied using the climate model CESM1.2.2 combined with the vegetation model BIOME4 and compared with that of the present day. The atmospheric dust loading obtained for these three periods is 35.9, 9.7 and 3.2 Tg, respectively. In comparison, the present-day dust loading is ∼24 Tg. It is found that the area of subtropical land is the major controlling factor in the variation of dust loading; the climate and the land plant evolution play a relatively minor role in these experiments where continental ice sheets are not considered and the reliability of BIOME4 in simulating vegetation of deep time is still uncertain. Although the simulated atmospheric dust loading in 240 Ma was much higher than that simulated for today, the fraction of dust deposited into the ocean (26%) was much lower due to its supercontinental configuration. Dust was able to cool the local annual mean surface temperature by a maximum of 2 °C in 240 Ma. A warming is obtained in the northern polar region in both 240 Ma and 130 Ma, likely due to strengthened meridional oceanic heat transport rather than due to snow darkening.

## Graphical abstract

**Figure:**
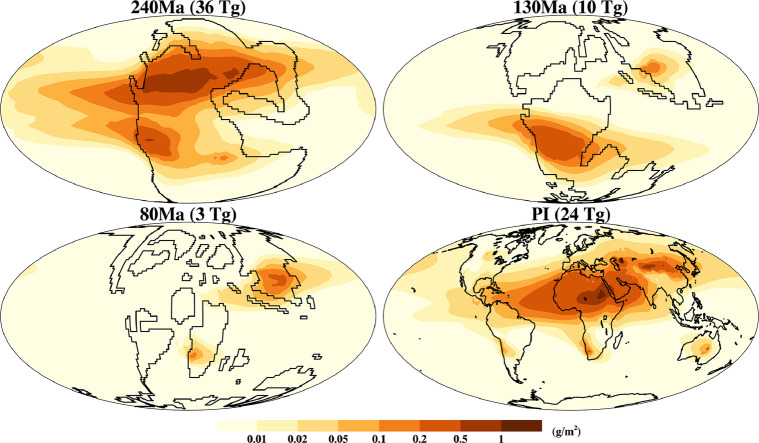

## 1 Introduction

Mineral dust, a primary natural aerosol, affects the Earth's climate and ecosystem in a variety of ways. Dust particles scatter and absorb the solar shortwave radiation, cooling the surface and warming the atmosphere [[1], [2], [3]]. They also absorb and emit long-wave radiation, warming the surface at night like greenhouse gases [[1], [2], [3]]. These are known as the direct radiative effect (DRE) of dust. Dust also interacts with clouds semi-directly through its thermal effect and indirectly by acting as cloud condensation nuclei, especially ice nuclei, just like other atmospheric aerosols [[2], [3], [4], [5], [6]]. Dust deposition on the snow- or ice-covered surface reduces the surface albedo, allowing the surface to warm up, known as the snow-darkening effect [[7], [8]]. Dust deposition to the surface can also provide nutrients, especially iron, to both terrestrial and marine ecosystems and perturb the biogeochemical cycles [[9], [10]]. Long-term deposition of eolian dust may have a profound influence on the surface morphology, the most prominent example being the formation of the Loess Plateau [11]. Despite its importance, a systematic understanding of the dust distribution in different time periods of the Earth is still lacking.

Throughout Earth's history, continental configuration, climate and vegetation have all changed dramatically [[12], [13], [14], [15]] and should impact on the dust cycle. For example, deserts tend to form in the subtropical region, meaning that continental drift could alter the area of desert and dust emission. During the cold Last Glacial Maximum (LGM; ∼21ka), both reconstructions and model simulations show that the dust loading and dust deposition rates were much higher than those during the interglacial period [[16], [17], [18], [19], [20], [21], [22], [23]]. Dust emission will change in the future too due to anthropogenic warming [24], but the results in the literature are mixed, with some indicating an increase [25] while others showing a decrease [26]. Previous modeling showed that the atmospheric dust loading could have been more than 10 times higher than today before land plants appeared [27]. During the past 250 Myr, land plants have evolved significantly; the hardier angiosperms first appeared in the Upper Triassic [28], and the more drought-tolerant C4 plants expanded and emerged in the Late Miocene [29]. Such changes of vegetation could have also impacted on the extent of desert and thus dust emission.

Here we plan to study the dust cycle during the past 250 Myr through numerical modeling by focusing on three typical periods only: 240 Ma, 130 Ma and 80 Ma. The reason to pick these three periods is because they had very different continental configurations (Fig. S1). The first time period was characterized by a supercontinent, the second by a mediumly fragmented continental configuration, while the third had the most fragmented continents during the past 250 Myr (see Fig. 4 of Hu et al. [30]). All three time periods had warm climate with the global mean surface temperature (GMST) all exceeding 20 °C [15], but sensitivity tests are carried out, mainly for the 240 Ma, to understand how the dust cycle is affected by climate. Sensitivity tests of land plant evolution are also performed. The aim is to obtain a sense of how the atmospheric dust has evolved during the past 250 Myr, and what roles the continental configuration, climate, and plant evolution might have played. Another goal is to have a rough understanding of how the dust has impacted on climate in the past.

Changes of the Earth's orbital configuration are also known to have a significant influence on the dust cycle. For example, the orbitally induced increase in summer solar insolation in the Northern Hemisphere (NH) during the early to mid-Holocene strengthened the monsoonal precipitation [31]. This change in precipitation led to the greening of the Sahara [[32], [33]] and a large reduction in dust emissions [[34], [35]]. Here we use the present-day orbital configuration and ignore its variation due to its relatively short timescale.

The paper is organized as follows. Section 2 describes the data, the model and the experimental setup. Section 3 presents the results, discusses the factors influencing the atmospheric dust loading and describes the impact of dust on climate. A summary is given in Section 4.

## 2 Methods

### 2.1 The climate model

This study uses the global coupled climate model, Community Earth System Model version 1.2.2 (CESM1.2.2) maintained by the National Center for Atmospheric Research (NCAR). The model consists of seven components: atmosphere, ocean, land, sea-ice, land ice, river and waves, connecting the components through a coupler [36]. The land ice and wave components are not activated in this study. The atmosphere (Community Atmosphere Model version 4, CAM4) and land (Community Land Model version 4, CLM4) components share the same horizontal grid (T31) with a resolution of 3.75° (zonal) x 3.75° (meridional). The atmosphere has 26 layers in the vertical direction. The ocean (Parallel Ocean Program version 2, POP2) and sea-ice (Community Ice Code 4, CICE4) share the same grid gx3v7, which uses a nominal 3° non-uniform horizontal grid. The ocean has 60 vertical layers. The river component (River Transport Model, RTM) has a resolution of 0.5° (zonal) x 0.5° (meridional).

The dust cycle consists of three parts: emission, transport and deposition [37]. The emission scheme follows the Dust Entrainment and Deposition (DEAD) model, which treats dust emission as a saltation-sandblasting process of particles under the influence of wind friction velocity (with a cubic dependence) [38], soil moisture, vegetation cover, snow cover, and soil erodibility [[39], [40]]. Dust emissions from the sandblasting process are represented by the vertical mass flux, which must be corrected for the erodible fraction of each model grid cell, i.e., the fraction of the area not covered by lakes, wetlands, snow, frozen soil and vegetation [41]. Based on the observations, the threshold of ground covered by vegetation leaves and stems is set at 0.3 m^2^/m^2^ [[37], [39]], above which the dust emissions are completely suppressed in the region. Certain areas of the Earth with high dust emissions have been observed to coincide with topographic depressions where large amounts of sediment are deposited [[42], [43], [44]]. These sediments are highly erodible, resulting in higher local dust emissions. The distribution of such regions is expressed by the soil erodibility factor *k*, which is multiplied by the vertical mass flux to correct for the amount of dust emitted to the atmosphere.

The transport and deposition of dust is simulated using the Bulk Aerosol Model (BAM) scheme. The size distribution is divided into four bins (0.1–1.0 µm, 1.0–2.5 µm, 2.5–5.0 µm, and 5.0–10.0 µm), with each fraction accounting for 3.8%, 11%, 17%, and 67% of the total mass flux at the emission [45]. During the transport and deposition processes, the proportion of dust in the four bins varies [21]. Only the shortwave direct effect is considered, that is, the longwave radiative effect of dust and the indirect effect of dust on clouds are not considered. The effect of dust on the biogeochemical cycle is not considered in the model either.

### 2.2 The vegetation model

**Fig. 1:**
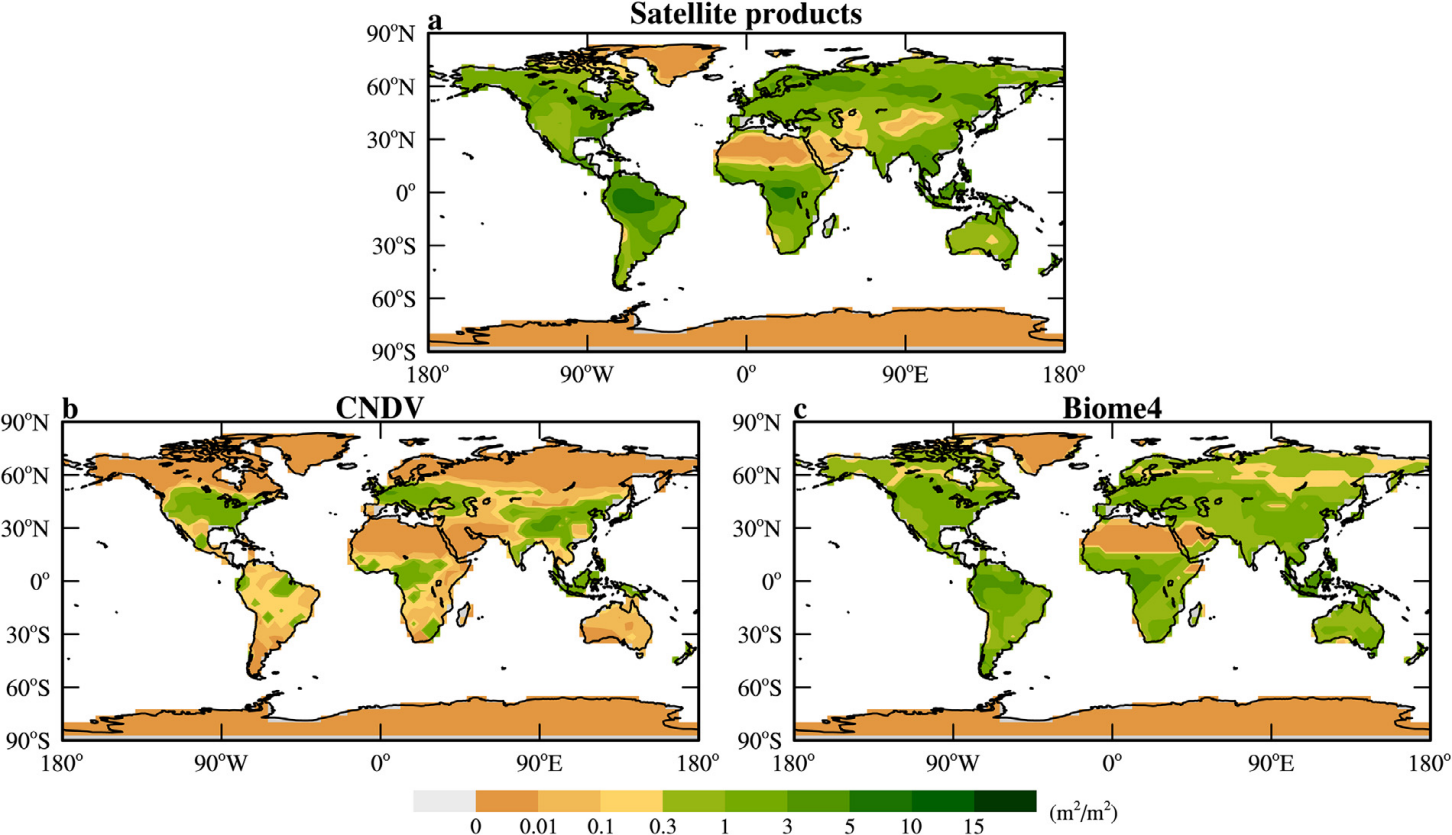
**PI vegetation cover (the sum of leaf plus stem area index) (m**^**2**^**/m**^**2**^**) from (a) satellite data, (b) CNDV simulation, and (c) BIOME4 simulation.**

The built-in carbon-nitrogen model with dynamic vegetation (CNDV) in the land module CLM4 of the CESM1.2.2 simulates an unreasonable present-day distribution of PFTs, significantly underestimating the tundra vegetation cover ([46]; see Fig. 1 here). As mentioned above in section 2.1, the fraction of vegetation cover over land is critical for dust emission, therefore, CNDV is clearly not a good choice for the current study. The coupled biogeographic and biogeochemical model, BIOME4, developed by Kaplan et al. [47] is used here instead. BIOME4 simulates the distribution of 28 biomes when atmospheric CO_2_ concentration (*p*CO_2_), monthly mean climate (precipitation, temperature, shortwave radiation), and soil physical properties (water holding capacity and infiltration rate) are provided. BIOME4 and its predecessors simulate paleo-vegetation well [[47], [48]] and have been used in many paleoclimate simulations [[21], [23], [37], [48], [49], [50]].

To couple BIOME4 with CESM1.2.2, they exchange information once every 40 years. When CESM1.2.2 is run for 40 years, the monthly precipitation, temperature, and shortwave radiation averaged over the last 10 years are input into BIOME4. *p*CO_2_ is also needed by BIOME4. Then BIOME4 is run to equilibrium, and the distribution of 28 biomes obtained are used to generate surface data in CESM1.2.2. This process is repeated until the simulation reaches quasi-equilibrium.

Inspection of Fig. 1 indicates that the vegetation simulated by BIOME4 is much closer to the observation than that simulated by CNDV and is therefore a more advisable choice than CNDV. It should be noted that BIOME4 tends to overestimate vegetation in arid regions such as Australia, Western and Central Asia but underestimate vegetation over the high-latitude regions (Fig. 1). This may lead to biases in the simulated dust as will be described in detail in the next section.

### 2.3 Soil erodibility

Soil erodibility *k* together with a universal emission factor affects the location of dust emissions and the amount of dust emitted to the atmosphere. In general, depressions have larger *k* values due to the accumulation of fine sediment transported from the upstream by surface runoff; the larger the upstream watershed area, the larger the *k* value [42]. Today's *k*-values vary between 0 and 5.7 (Fig. S3), with a global average of 0.14 at the horizontal resolution T31 described above (this value is resolution-dependent in the model, the higher the resolution, the smaller the value), resulting in a total simulated atmospheric dust of 24 Tg. However, in other periods of the Earth's geological history, it is difficult to grasp the details of topographic features and to determine the spatial distribution of soil erodibility. Therefore, a globally uniform *k* value is used in this paper.

**Fig. 2:**
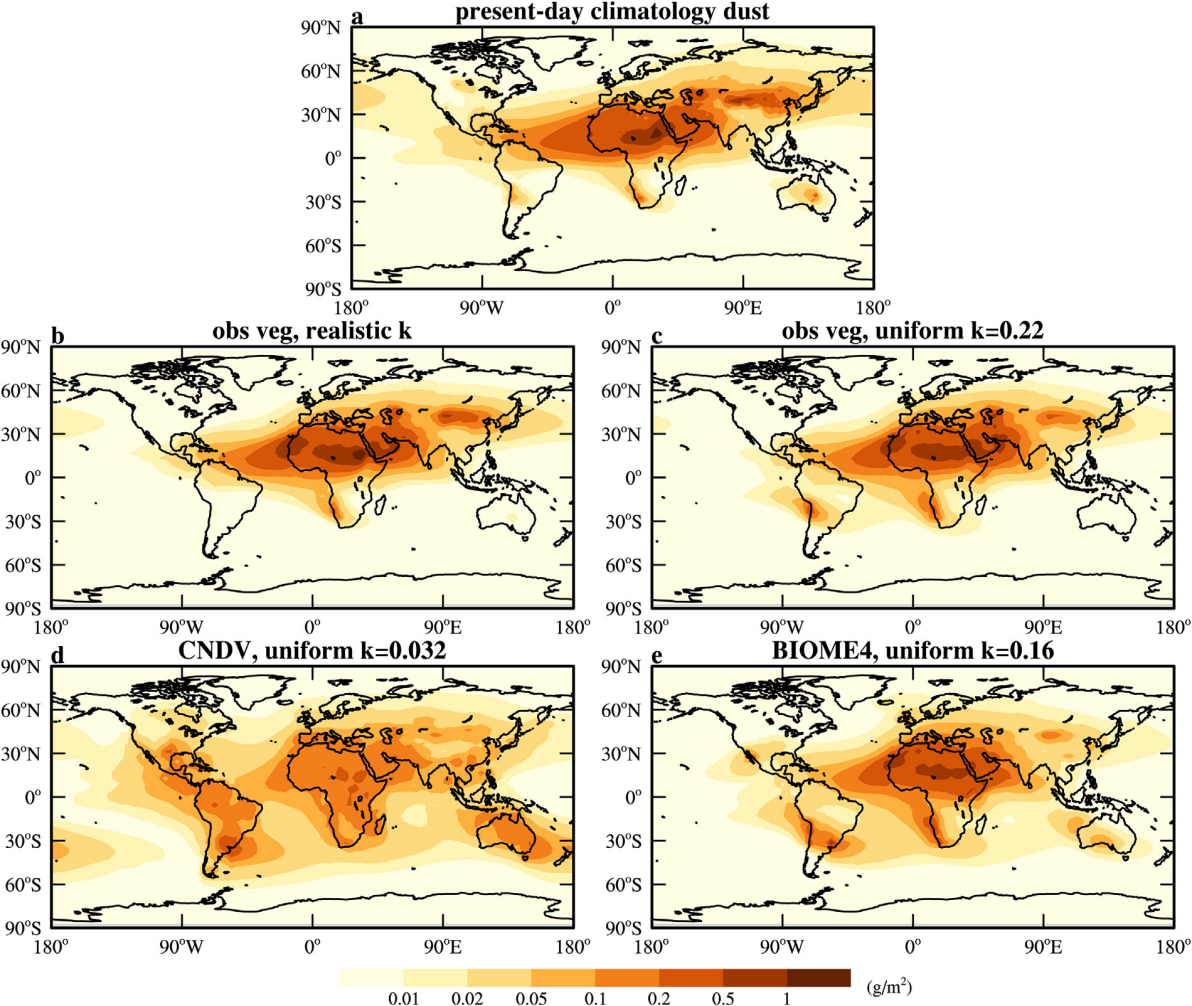
**Annual-mean atmospheric dust loading (g/m^2^) for PI.** (a) Present-day climatological soil dust aerosols obtained from chemical transport model forced with meteorological reanalysis data and constrained by assimilation of satellite aerosol retrievals [45]. Simulation results obtained in CESM1.2.2 by using the (b) observed vegetation and the geomorphic soil erodibility [[21], [39]], (c) observed vegetation and a globally uniform *k* of 0.22, (d) CNDV simulated vegetation and a globally uniform *k* of 0.032, and (e) BIOME4 simulated vegetation and a globally uniform *k* of 0.16. The global dust loading is 24 Tg in all simulations.

The universal emission factor is first tuned to make sure that the simulated present-day (taken as a surrogate for the pre-industrial) global dust loading is correct when the observed vegetation distribution and realistic *k* are used (see the result in Fig. 2b). Then, we test whether a uniform *k* would produce a reasonably well atmospheric dust loading when the observed vegetation is prescribed. The result shows that the spatial pattern of the dust loading is quite similar to that of the realistic one and the total loading is almost the same as the observed one when *k* is set to 0.22 (compare Fig. 2c with a). Then, we further test whether a uniform *k* combined with the model-simulated vegetation would also produce a reasonable dust loading. It is found that pattern of dust loading is very different from the observed one if CNDV is used to simulate vegetation (compare Fig. 2d with a). While the simulated dust loading is quite good when the BIOME4 is used to simulate vegetation, for which the value of 0.16 needs to be used.

Fig. 2 shows that errors in the simulated climate, vegetation, and surface erodibility all induce biases in the simulated dust loading. The influence of the error in climate can be inferred by comparing Fig. 2b to a. Even if the observed vegetation distribution and realistic *k* are used, the simulated dust loading as well as emission (not shown but can be easily deduced from the dust loading) is significantly underestimated in the Southern Hemisphere (SH) and the mid- to high-latitude NH. This is mainly due to the overestimation of soil moisture in these regions (not shown).

The influence of using a globally uniform *k* can be obtained by comparing Fig. 2c to b. When the realistic *k* is replaced by the uniform *k*, dust emission over South America and southern Africa is enhanced but that in the central Asia is reduced. This is unsurprising since the uniform *k* value (0.22) is larger than the realistic *k* in many regions (Fig. S3) and is also larger than the global mean of realistic *k* (0.14). Therefore, using a uniform k, to some extent, compensates the biases due to inaccurate model-simulated climate, but in the meantime smooths out the peak emission at some hotspots such as in North Africa and central Asia.

The influence of errors in the vegetation simulated by BIOME4 can be inferred by comparing Fig. 2e to c. There is generally too little vegetation over the high-latitude region, but dust emission is not overestimated there (except over Iceland and United Kingdom) because of the overestimated soil moisture as mentioned above. Too much vegetation is simulated over western and central Asia, and thus dust emission is highly underestimated. The vegetation distribution in the SH is simulated quite well (Fig. 1). However, even the error in a single grid point within the subtropical region may produce a significant bias in dust emission. For example, the vegetation is underestimated in a grid point on the eastern coast of the southern South America gives off large amount of dust (Fig. 2e). The same also happens in North America but not as severe.

Therefore, a globally uniform *k* combined with BIOME4-simulated vegetation tends to overestimate dust emission in the subtropical region but underestimate dust emission over hotspot regions. The model also tends to underestimate dust emission over regions over or surrounded by high topography such as Iranian and Tibetan Plateau. These features may hold for other time periods focused by this study. Nonetheless, the overall pattern of dust loading is quite similar to that of the observation (compare Fig. 2e to a) which gives us confidence in applying this strategy to modeling dust cycle of deep-time Earth. For the deep-time Earth, another source of error is present; there may be large uncertainties in the reconstructed land-sea mask and surface topography.

The modeling strategy leads to more dust being produced near the coast, resulting in a larger fraction of the dust being deposited over the ocean. For example, the fraction of oceanic dust deposition corresponding to Figs. 2c and e is ∼38% and 44%, respectively, which is much higher than that estimated in Fig. 2b (33%), and in Albani (16%) [10] and Jickells (26%) [9]. Another reason that may be responsible for the overestimated oceanic dust deposition is the size distribution of the dust used here; the proportion of fine dust (the 0.1–1.0 µm and 1.0–2.5 µm bins) occupies 14.8% by volume of the total dust emitted here, but it was only 9.8% in Kok [51] and Albani [10]. The fine dust is much more easily subject to long-distance transport [37], thus increasing dust deposition to the ocean.

### 2.4 Experimental setup

For continental configurations, those reconstructed by Scotese and Wright [14] are used (Fig. S1). The boundary conditions for all experiments performed herein are from the corresponding experiments in Li et al. [52]. Aerosols, including dust, in their simulations were given a globally uniform distribution, and vegetation was simulated using CNDV embedded in CESM1.2.2. Continental ice sheets are not simulated in any of the experiments except in the PI simulations where the present-day ice sheets are prescribed (i.e., the ice sheets are present but static). The solar constant was reduced at a rate of 0.08% per 10 Myr relative to today [53]. They adjusted the *p*CO_2_ for each time slice so that the simulated GMST was within 0.5 °C of that reconstructed one by Scotese [54]. The final *p*CO_2_ they obtained for 240 Ma, 130 Ma and 80 Ma was 7840 ppmv, 2520 ppmv and 1960 ppmv, respectively. These *p*CO_2_ are quite high compared to reconstructed values [55], mainly due to the deficiency of CNDV which produces too little vegetation and induces a strong cooling effect. Here we re-do the experiments (240Ma_7840CO_2__7840Veg, 130Ma_2520CO_2__2520Veg, 80Ma_1960CO_2__1960Veg in Table 1) by coupling CESM and BIOME4. These experiments are continued for 4000 years and are considered as control experiments. The GMSTs obtained for the three periods are 3 °C, 3 °C, and 2 °C higher than those in Li et al. [52]. Given that the climate sensitivity of CESM1.2.2 is 2.9 °C, this means that *p*CO_2_ for 240 Ma and 130 Ma in Li et al. [52] could have been halved if BIOME4 had been used. The warming effect of vegetation can also be seen clearly in the four experiments carried out for PI (Table 1) and has been systematically studied by Guo et al. [56].

**Table 1:** **Summary of the experiments carried out in this study.** All the experiments listed below are in pairs, one without dust and the other with dust.

	Experiment	*p*CO_2_ (ppmv)	Vegetation setup	Dust emission (Tg/yr)	Dust loading (Tg)	Dust deposition over ocean (Tg/yr)	GMST ( °C)
1	240Ma_7840CO_2__7840Veg	7840	ang., C4	1956.7	35.9	509.6 (26%)⁎⁎	28.5 (28.6)⁎⁎⁎
2	130Ma_2520CO_2__2520Veg	2520	ang., C4	587.4	9.7	154.8 (26%)	24.3 (24.3)
3	80Ma_1960CO_2__1960Veg	1960	ang., C4	246.1	3.2	107.3 (44%)	26.3 (26.4)
4	240Ma_2240CO_2__7840Veg	2240	ang., C4	1842.4	30.1	463.1 (25%)	21.8
5	240Ma_1120CO_2__7840Veg	1120	ang., C4	1863.6	27.4	454.3 (24%)	18.3
6	240Ma_560CO_2__7840Veg	560	ang., C4	1934.2	26.3	459.0 (24%)	14.4
7	240Ma_280CO_2__7840Veg	280	ang., C4	1988.5	25.0	449.5 (23%)	10.7
8	240Ma_2240CO_2__2240Veg	2240	ang., C4	1315.8	21.1	347.7 (26%)	21.7 (21.8)
9	240Ma_1120CO_2__1120Veg	1120	ang., C4	1217.0	18.5	360.4 (29%)	18.1 (18.2)
10	240Ma_560CO_2__560Veg	560	ang., C4	2272.9	30.2	494.3 (22%)	9.6 (9.4)
11	240Ma_280CO_2__280Veg	280	ang., C4	3155.0	37.1	655.6 (21%)	4.4 (4.4)
12	240Ma_7840CO_2__NoC4	7840	ang., no C4	1872.7	35.3	470.0 (25%)	28.6 (28.7)
13	240Ma_7840CO_2__NoAng	7840	no ang., C4	1921.1	36.4	475.1 (25%)	28.7 (28.8)
14	240Ma_7840CO_2__NoAngNoC4	7840	no ang.,no C4	1765.4	33.2	464.0 (26%)	28.8 (28.9)
15	PI_280CO_2__VegObs_k*	280	ang., C4	1866.0	23.7	624.3 (33%)	13.0
16	PI_280CO_2__VegObs	280	ang., C4	1905.5	23.5	727.6 (38%)	13.1
17	PI_280CO_2__VegCNDV	280	ang., C4	2766.9	24.4	1343.9 (48%)	10.4
18	PI_280CO_2__VegBIOME4	280	ang., C4	2217.8	23.7	991.3 (44%)	12.0

⁎ The surface erodibility with realistic geographic distribution is used only in this experiment.

⁎⁎ The fraction of dust deposition over the ocean to total global deposition (equal to the gobal emission) is given in brackets.

⁎⁎⁎ The inner and outer brackets represent the global mean surface temperature of the dust experiment and the non-dust experiment respectively.

In order to distinguish the effects of continental configuration and climate on the dust cycle, two sets of sensitivity experiments are performed for 240 Ma. Each set consists of four experiments with *p*CO_2_ lowered to 2240 ppmv, 1120 ppmv, 560 ppmv, and 280 ppmv, respectively. The two sets of experiments differ in whether the vegetation responds to the climate change; in one set, the same vegetation as in experiment 240Ma_7840CO_2__7840Veg is used, while in the other, vegetation is equilibrated with the new *p*CO_2_ and climate. The experiments in the first set are named as 240Ma_2240CO_2__7840Veg and 240Ma_1120CO_2__7840Veg, etc., and those in the second set are named as 240Ma_2240CO_2__2240Veg and 240Ma_1120CO_2__1120Veg, etc. (Table 1). These experiments are carried out for at least 2000 years.

By default, the angiosperms and C4 plants are included in all experiments. To test the influence of vegetation evolution on the dust cycle, another set of three sensitivity experiments are run with the same *p*CO_2_ as in the experiment 240Ma_7840CO_2__7840Veg. In these sensitivity experiments, C4 plants, angiosperms, and both angiosperms and C4 plants are removed, and are named as 240Ma_7840CO_2__NoC4, 240Ma_7840CO_2__NoAng, and 240Ma_7840CO_2__NoAngC4, respectively (Table 1). These experiments are also carried out for at least 2000 years.

To obtain the dust cycle as well as the impact of dust on climate, branch runs are continued from the equilibrium state of all the experiments above (Experiments 1–18 in Table 1). In these branch runs, dust cycle is activated but the vegetation does not change anymore. These branch runs share the same experiment names from which they are branched off; thus, each experiment in Table 1 actually contains two runs, one without dust and the other with dust. These branch runs are continued for at least 600 years. For all the simulations described above, the last 100 years of data are used for analyses.

### 2.5 Reconstruction data

**Fig. 3:**
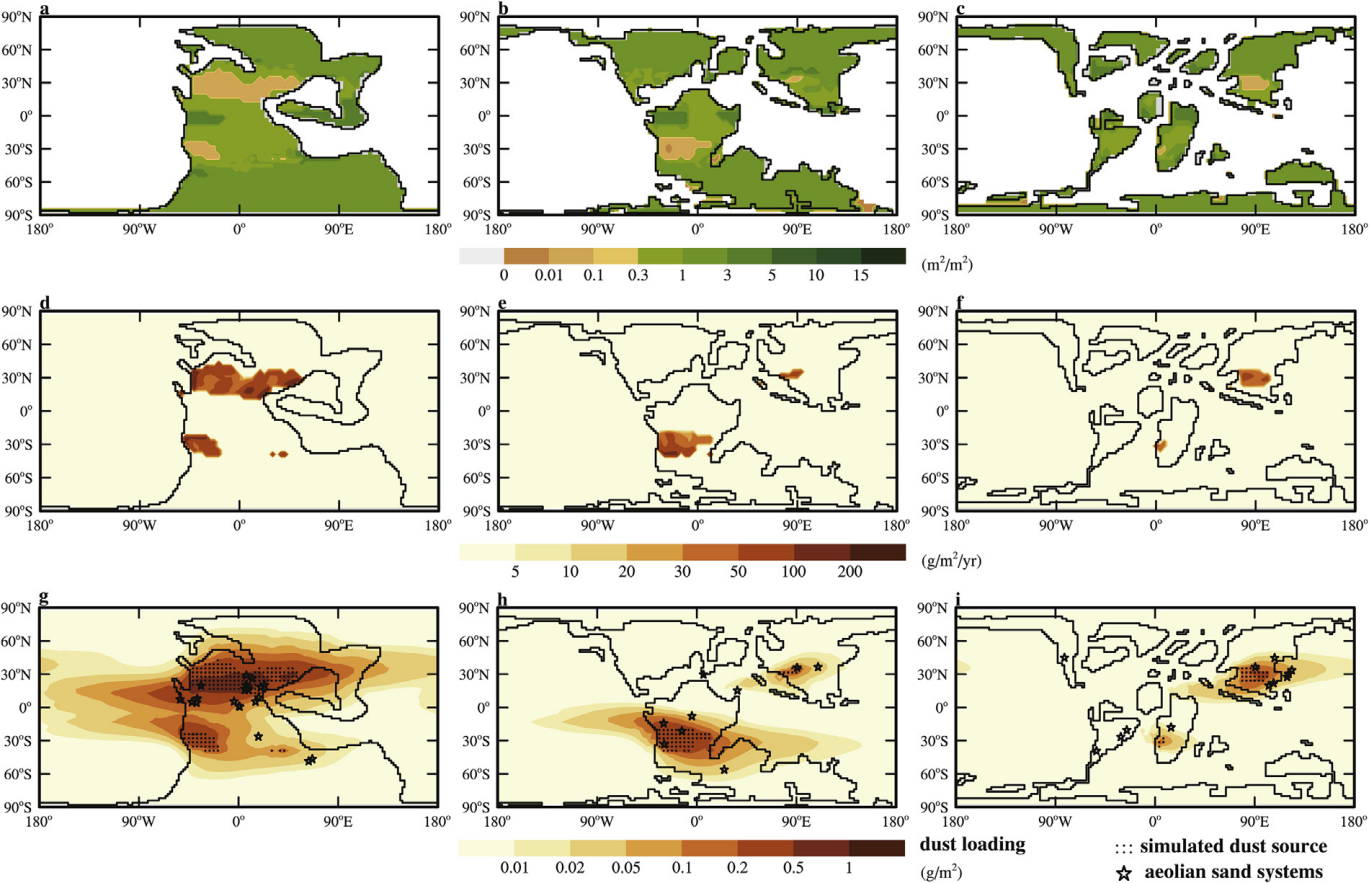
(a-c) Vegetation cover (the sum of leaf plus stem area index) (m^2^/m^2^) simulated by Biome4. (d-f) Annual-mean dust emission (g/m^2^/yr). (g-i) The comparison between the simulated dust cycles for dust loading (color-filled contours, g/m^2^), dust source (dots) and the sedimentary record of aeolian sand systems (stars). (a,d,g) 240Ma_7840CO_2__7840Veg, (b,e,h) 130Ma_2520CO_2__2520Veg, (c,f,i) 80Ma_1960CO_2__1960Veg.

To evaluate the simulated dust cycle, dust-related geological records are compiled from the literature for the three time periods. Specifically, paleo-locations of aeolian sand systems and loess sites are collected. The aeolian sand systems are mostly inland and coastal sand dune systems [57], indicating that this area may have been a desert, dune, or sandy area in the past. Due to the very limited records that can be found, all the records for the whole Triassic, Early and Late Cretaceous are treated as 240 Ma, 130 Ma and 80 Ma, respectively. Widespread desert conditions prevailed across much of Europe during the Triassic, close to the eastern margin of Northern Pangea [[57], [58], [59], [60], [61], [62], [63], [64], [65], [66], [67], [68], [69], [70]]. Most of the other records are from North America, particularly the southwestern United States [[57], [71], [72], [73], [74], [75], [76], [77], [78]], and only three records are found in the SH [[57], [79], [80]]. During the Cretaceous, aeolian deposits are widespread in Asia [[81], [82], [83], [84], [85], [86], [87]], South America [[57], [88], [89], [90], [91], [92], [93], [94], [95], [96]] and Africa [[97], [98], [99]]. The paleo-locations of all the records are shown in Fig. 3g-I and listed in Table S1.

## 3 Results and discussion

### 3.1 Simulated dust for 240 Ma, 130 Ma, and 80 Ma

The total dust loading simulated for 240 Ma is about 1.5 times the PI atmospheric level (PAL; 24 Tg) and decreases monotonically to 0.4 times PAL and 0.13 times PAL for 130 Ma and 80 Ma, respectively (Table 1). The spatial pattern of dust loading is determined by the spatial distribution of the vegetation (Fig. 3a-c) and the winds (Fig. S4a-c). For 240 Ma, vegetation cannot grow within wide areas of the subtropical regions (Fig. 3a), especially in the NH (Fig. 3d). Such regions become progressively smaller towards 80 Ma (Fig. 3b,c) and then widen again significantly towards the present day (Fig. 1). The regions of heavy dust loading simulated for the three time periods correspond well with the sand records for the Triassic, Early Cretaceous and Late Cretaceous, respectively (Fig. 3g-i). In some regions of 130 Ma and 80 Ma, records indicate presence of dust deposition, but no dust loading or deposition is simulated. This misfit is especially severe for 80 Ma. Since the modeling strategy in the current study tends to overestimate the extent of dust source area within the subtropical region, the underestimate of dust emission in these cases might be due to uncertainty in the continental reconstruction.

**Fig. 4:**
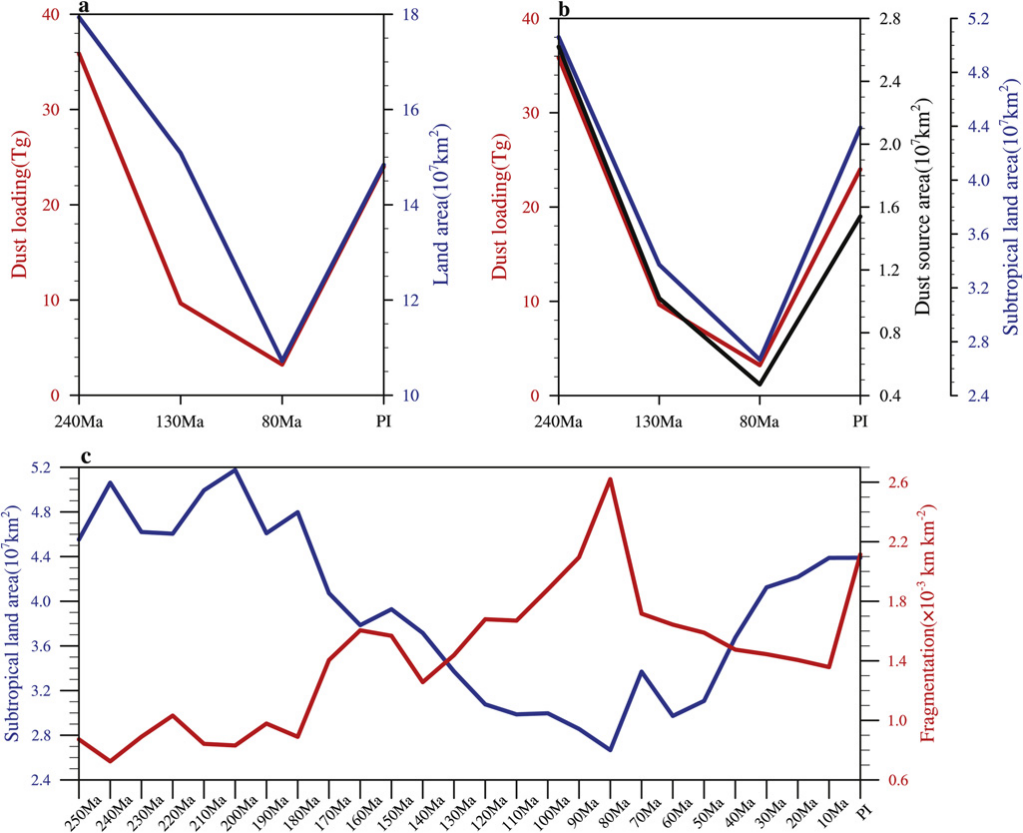
**The relationship between global annual-mean atmospheric dust loading and (a) land area, (b) subtropical land area and dust source area.** The subtropical land area during the past 250 Myr is shown in (c). The subtropical region is defined as the area between 20° and 40° latitude in both hemispheres.

The temporal variation of the dust loading is clearly related to the evolution of the continental configuration. Especially, the land area within the subtropical region seems to have the greatest influence, since it is where deserts are predominantly formed (Fig. 3). Indeed, the simulated dust loading covaries with the subtropical land area (Fig. 4b), better than with the total land area (Fig. 4a). For the same total land area within the subtropical region, the continental fragmentation may also have some influence on the area of desert and thus on the dust cycle, since smaller land may have easier access to water vapor than larger land. Unfortunately, this cannot be tested with the above experiments because the fragmentation of Pangea, the latitudinal shift of the continental pieces and the rise in sea level all happened at the same time ([30]; a measure of the fragmentation from them is put in Fig. 4c). The co-occurrence of these events makes it difficult to say whether the continental fragmentation played a significant role in reducing the dust loading from 240 Ma to 80 Ma. If the subtropical land area controls the dust loading, then we may infer from the evolution of this parameter (Fig. 4c) that the global dust loading was probably much less than it is today for most time of the past 250 Myr. Note that this inference is very crude because a robust relationship cannot be established from four data points in Fig. 4b.

Dust deposited into the ocean is an important source of nutrients, especially iron [[9], [10], [22], [100]]. Such fluxes are 509.6 Tg/yr, 154.8 Tg/yr, and 107.3 Tg/yr for 240 Ma, 130 Ma and 80 Ma, respectively (Table 1). All these fluxes are lower than or near that in the more properly setup PI experiment (440 Tg/yr) [10], and much lower than that simulated here using a spatially uniform *k* for PI (991.3 Tg/yr, corresponding to Fig. 2e). These fluxes represent 26%, 26% and 44% of the corresponding emissions. Again, a robust relationship cannot be obtained but Fig. 3 seems to indicate that smaller subtropical continents would allow larger fractions of dust to deposit into the ocean. Physically, the pattern also makes sense because dust in a supercontinent would have to travel a farther distance to reach the oceans.

### 3.2 Impact of climate evolution

Other than the continental configuration, the climate has also evolved over the past 250 Myr (see Section 1). It is unclear whether the very different climates at different time periods contributed significantly to the change of the dust cycle. Therefore, we decrease the *p*CO_2_ in 240 Ma to see if the cooling of the climate in this case significantly changes the dust emission and loading. When *p*CO_2_ is lowered to 2240 ppmv, 1120 ppmv, 560 ppmv and 280 ppmv, the GMST is decreased to 21.7 °C, 18.2 °C, 9.4 °C and 4.4 °C, respectively (Table 1). The simulated impact on dust emission depends on whether vegetation responds to the climate, as will be described in detail below.

**Fig. 5:**
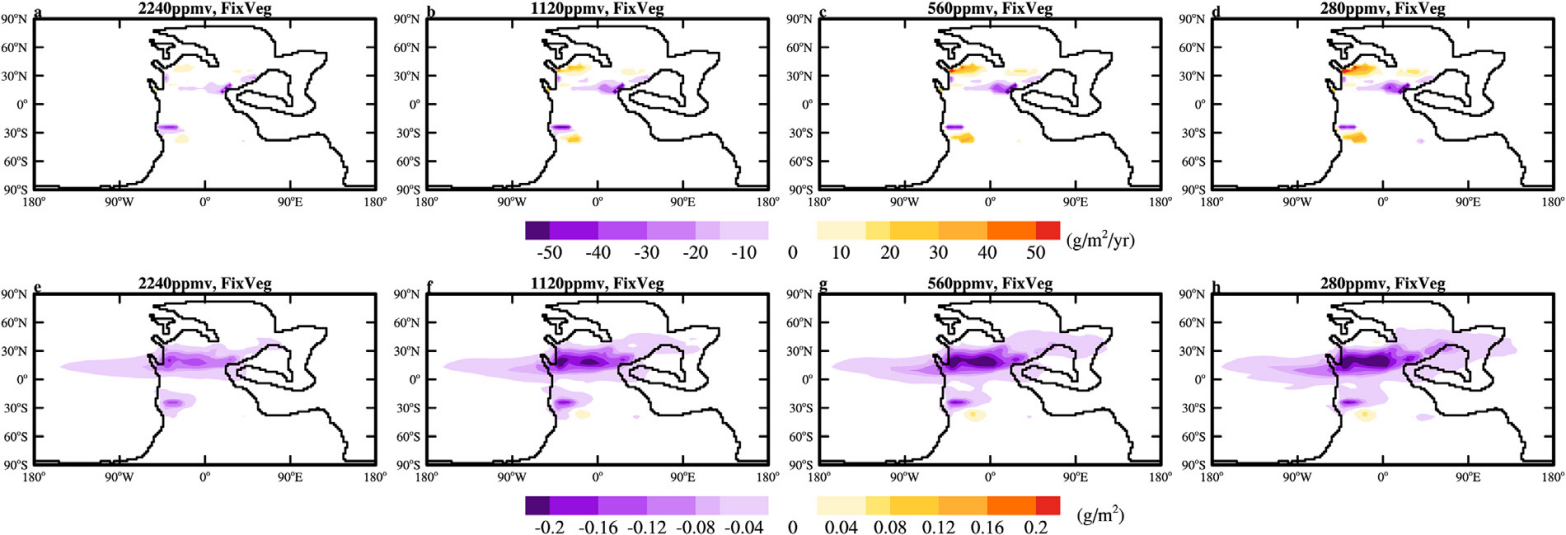
**The changes in the annual-mean (a-d) dust emission (g/m^2^/yr) and (e-h) atmospheric dust loading (g/m^2^) relative to 240Ma_7840CO_2__7840Veg when *p*CO_2_ is lowered to (a,e) 2240 ppmv, (b, f) 1120 ppmv, (c, g) 560 ppmv and (d, h) 280 ppmv.** The vegetation cover is fixed to that of 240Ma_7840CO_2__7840Veg.

**Fig. 6:**
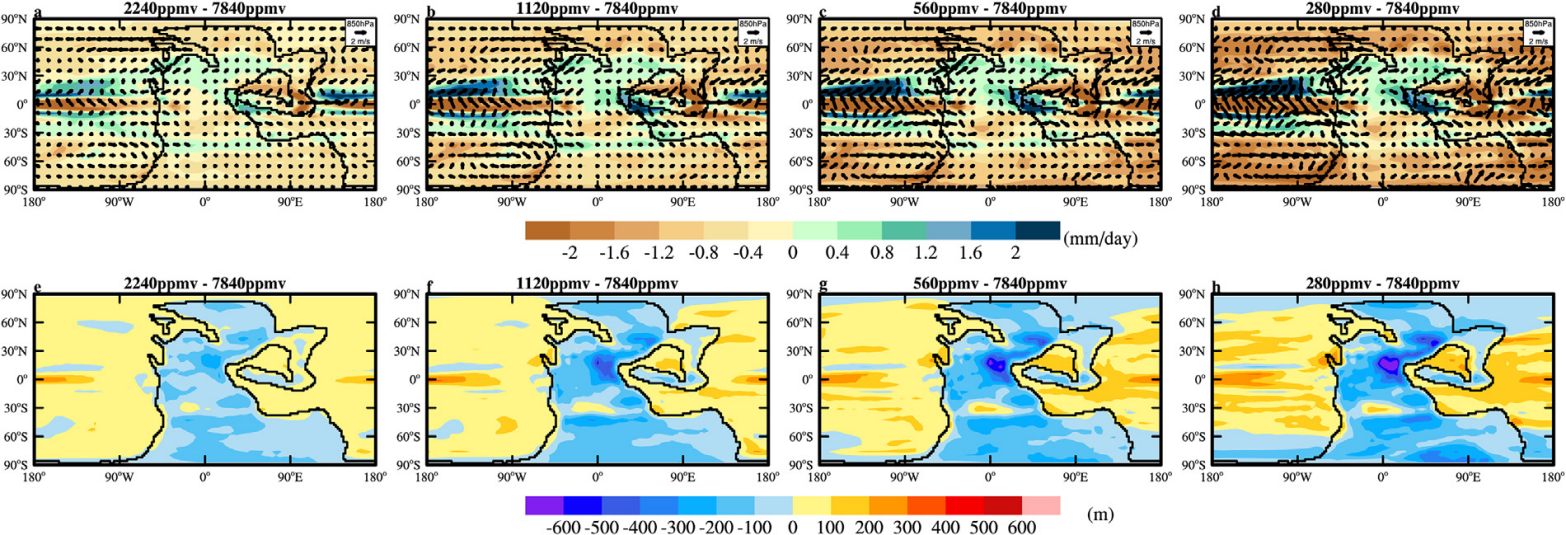
**The changes in annual-mean (a-d) precipitation (mm/day; colors) and 850 hPa wind (m/s; arrows) and (e-h) planetary boundary layer height (m) relative to 240Ma_7840CO_2__7840Veg under different *p*CO_2_.** The vegetation cover is fixed to that of 240Ma_7840CO_2__7840Veg.

When the vegetation is fixed to that (Fig. 3a; experiment 240Ma_7840CO_2__7840Veg) simulated by BIOME4 under the default *p*CO_2_ for 240Ma, the dust emission decreases from 1956.7 Tg/yr to 1842.4 Tg/yr and 1863.6 Tg/yr when the *p*CO_2_ is lowered to 2240 ppmv and 1120 ppmv, while increases to 1934.2 Tg/yr and 1988.5 Tg/yr when the *p*CO_2_ is lowered to 560 ppmv and 280 ppmv, respectively (experiments 4–7 in Table 1). Dust emission generally increase in the western mid-latitudinal regions and decrease in the eastern relatively lower-latitudinal regions (Fig. 5a-d). The increase is due to the strengthening of surface wind between 30° and 40° (Fig. 6a-d) while the decrease is due to increased precipitation and thus soil moisture in the east as the climate cools (Fig. 6a-d). The increase in the west and decrease in the east largely cancels each other, making the change of total emission small.

Surprisingly, the atmospheric dust loading decreases monotonically, from 35.9 Tg to 30.1 Tg, 27.4 Tg, 26.3 Tg and 25.0 Tg when *p*CO_2_ is lowered to 2240 ppmv, 1120 ppmv, 560 ppmv and 280 ppmv, respectively (Table 1; Fig. 5e-h). Therefore, the cooling must have somehow shortened the lifetime of the dust. The wet deposition is found to be the key reason for this shortening; despite a decreasing trend in dust emission in the eastern part of the continent (Fig. 5a-d), the wet deposition increases with the cooling (Fig. S5f-j). The strengthening of wet deposition is again consistent with the enhanced precipitation near the dust emission region (Fig. 6a-d). The shortening of the dust lifetime may also be related to the lowering of the atmospheric boundary layer as the climate cools (Fig. 6e-h), but this is difficult to quantify.

**Fig. 7:**
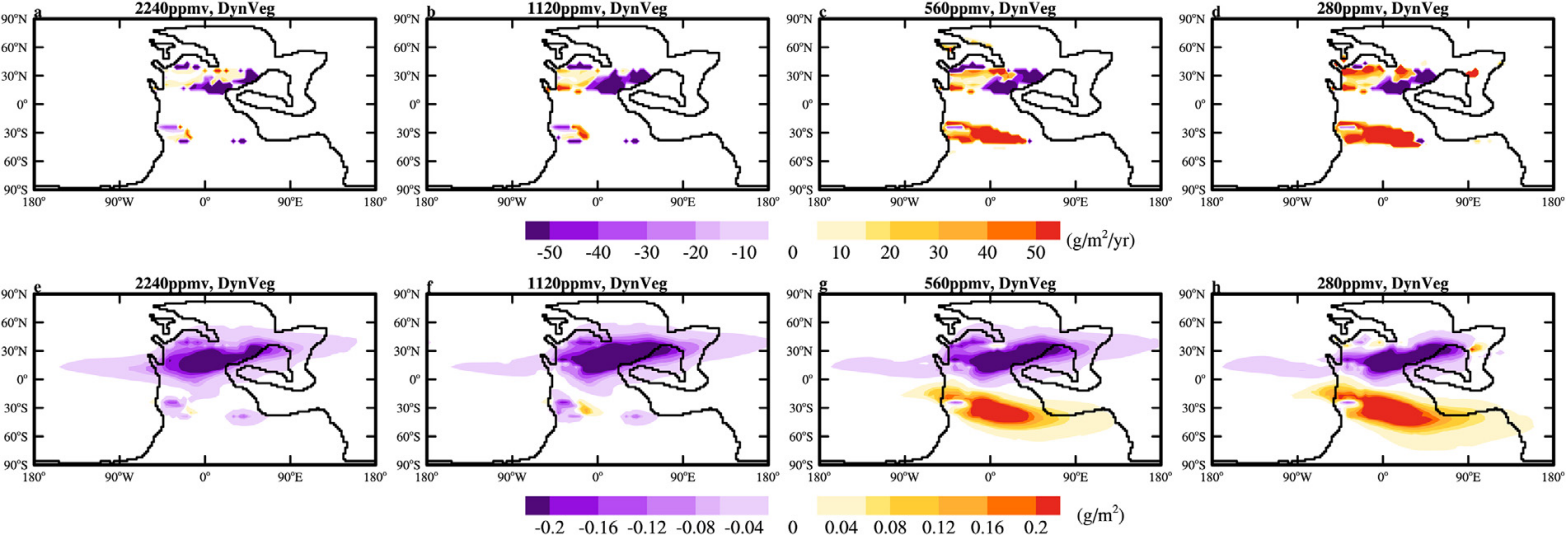
**The changes in the annual-mean (a-d) dust emission (g/m^2^/yr) and (e-h) atmospheric dust loading (g/m^2^) relative to 240Ma_7840CO_2__7840Veg under different *p*CO_2_.** The vegetation cover is dynamically updated with changes in climate and *p*CO_2_.

When vegetation is dynamically updated during the cooling (experiments 8–11), both dust emission and dust loading decrease substantially first and then increase as the climate cools (Table 1). The decrease occurs predominantly in the NH, similar to that in experiments with fixed vegetation but with larger magnitude (compare Fig. 7 to Fig. 5). The larger magnitude is due to the expansion of vegetation and thus the retreat of deserts (Figs. S6). The reduction in dust emission in the NH is the dominant phenomenon when *p*CO_2_ is lowered to 2240 ppmv and 1120 ppmv. When *p*CO_2_ is lowered further, the dust source region in the SH expands substantially (Figs. 7c, d and S6d, e, i, j), resulting in a noticeable increase in dust loading (Fig. 7g, h). In the coldest case (experiment 240Ma_280CO_2__280Veg), the dust emission increases by more than 60% compared to the control case (experiment 240Ma_7840CO_2__7840Veg). However, even in this case, the dust loading is only 37.1 Tg, comparable to that (35.9 Tg) in the control case (Table 1). This again indicates a significant shortening of the lifetime of dust. Note that when *p*CO_2_ is lowered to 280 ppmv, large areas of bare land appear in the polar region (Fig. S6e,j), but these are covered by snow and no dust emission is allowed.

### 3.3 Impact of land plant evolution

**Fig. 8:**
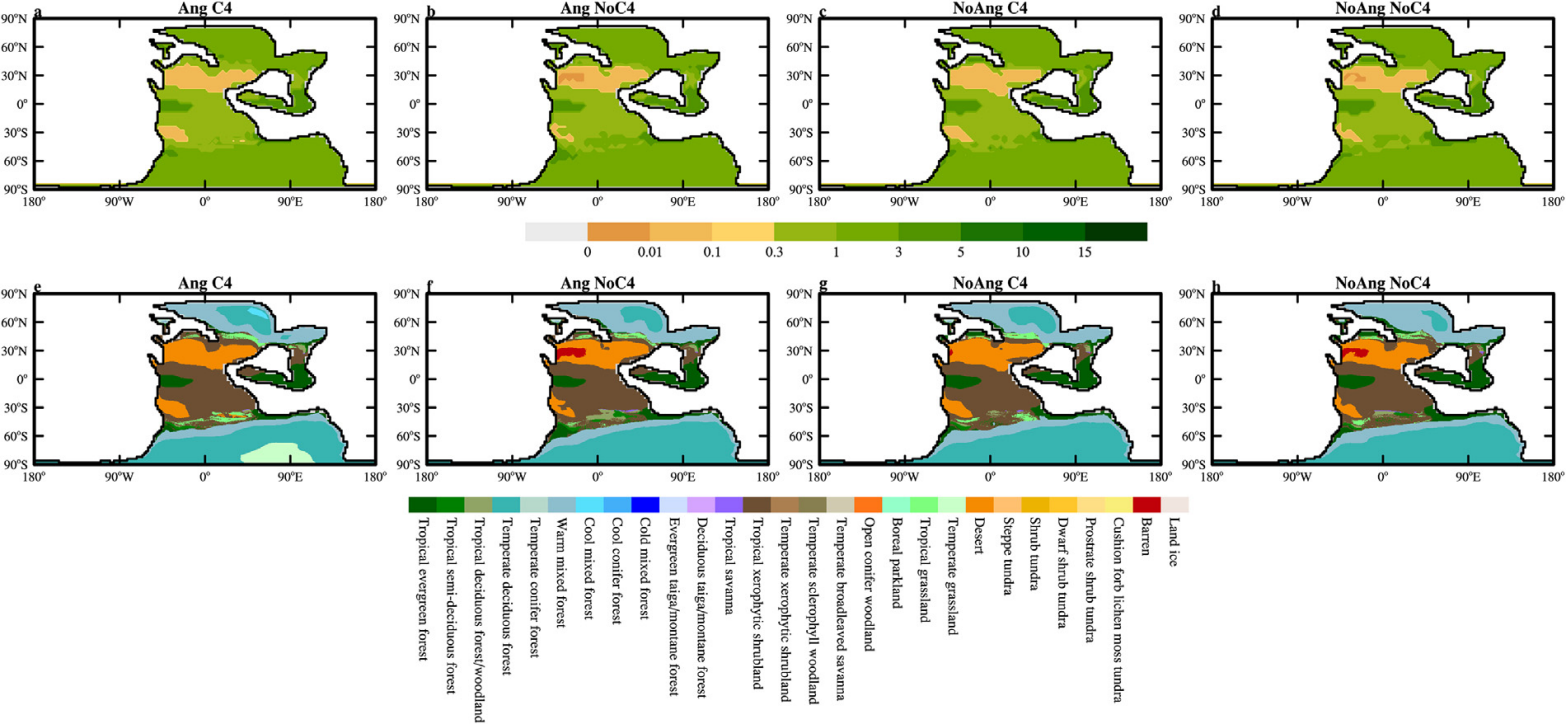
(a-d) Vegetation cover (the sum of leaf plus stem area index) (m^2^/m^2^) and the corresponding (e-h) biomes map simulated by BIOME4 for 240 Ma using different vegetation settings. There are both angiosperms and C4 plants in the left most column, and C4 plants and angiosperms are removed in the second and third columns, respectively. In the right most column, there is neither angiosperms not C4 plants.

Simulation results show that the evolution of land plants has a complex influence on the distribution of dust source; the appearance of the supposedly more dryness-resistant and coldness-resistant angiosperms and C4 plants does not necessarily reduce the area of deserts (Fig. 8). When angiosperms are not allowed to grow, tropical xerophytic shrublands shrink towards the North Pole (compare Fig. 8g to e), slightly increasing the dust emission and loading. When C4 plants are not allowed to grow, part of the NH desert interior becomes completely barren and the northern boundary expands northward; while part of the SH desert becomes occupied by tropical xerophytic shrublands (compare Fig. 8f to e). Because barren land emits dust similarly to desert in the model, the consequence is that the global dust emission decreases slightly. When both angiosperms and C4 plants are not allowed to grow, it turns out that the gymnosperms grow just fine and the desert area actually shrinks (more significant in SH; compare Fig. 8h to e). The global dust emission decreases slightly (Table 1). Such changes in the distribution of vegetation are surprising, and may be due to unknown deficiency in the vegetation model. As a result, the impact of the land plant evolution on the dust cycle can be ignored but future investigation using other vegetation model is needed to make sure of this.

### 3.4 Impact of dust on climate

**Fig. 9:**
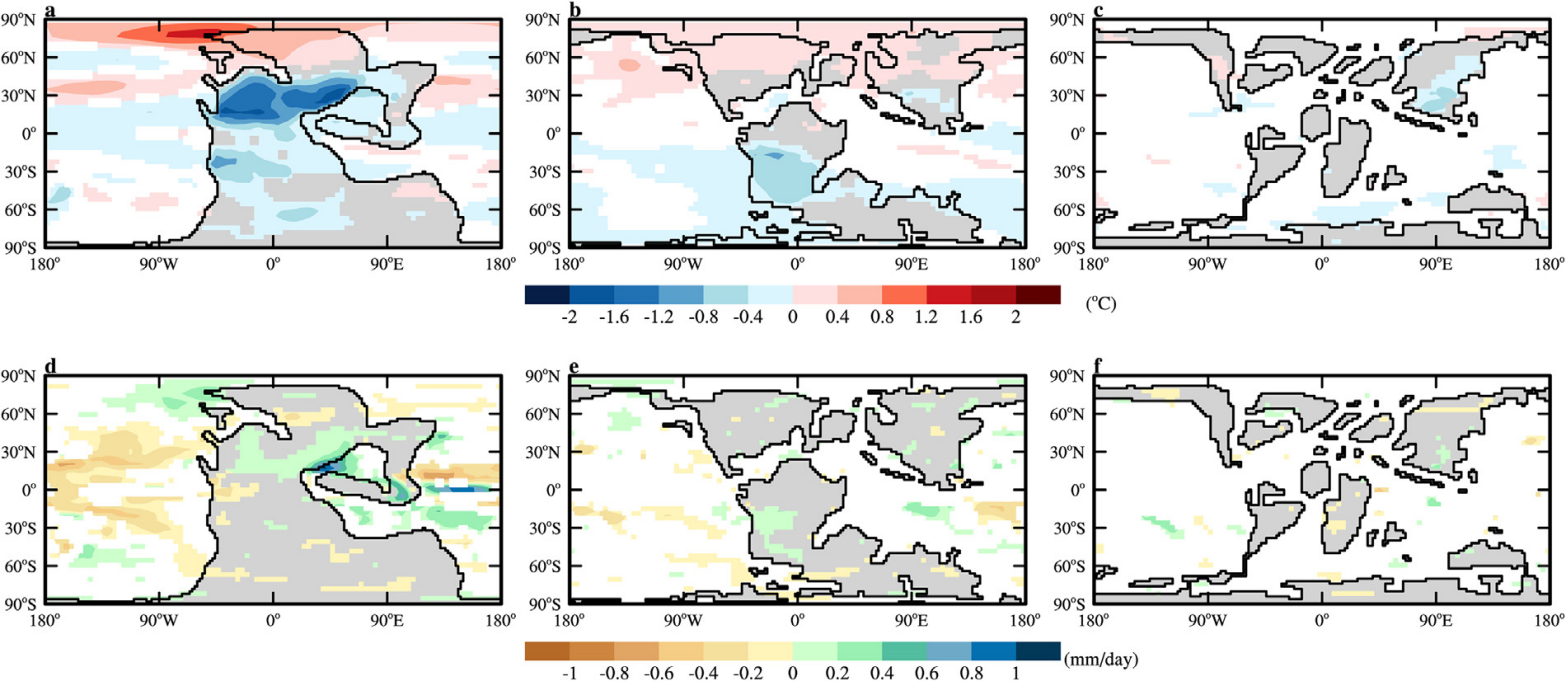
**The changes of annual-mean (a-c) surface temperature (**°**C) and (d-f) precipitation (mm/day) due to dust.** (a, d) 240Ma_7840CO_2__7840Veg, (b,e) 130Ma_2520CO_2__2520Veg, (c,f) 80Ma_1960CO_2__1960Veg. Only the changes that are significant to 0.1 level are shown.

Since the longwave warming effect and indirect effect (through cloud) of dust are not considered in the experiments carried out herein, the main effect of dust is to cool the surface by scattering, reflecting, and absorbing incoming sunlight. Note that because dust absorbs sunlight and warms the atmosphere, it could warm the surface by various processes if the amount of dust is huge [101]. In all experiments here, the cooling effect is dominant and is most significant near the dust source regions where the dust loading is high (Fig. 9a-c). For the three periods of the Mesozoic tested here, the 240 Ma dust clearly has the largest impact on the surface climate, as it has the largest dust loading; the local annual mean surface temperature is reduced by up to 2.0 °C (Fig. 9a). The maximum local cooling is 1.0 °C for 130 Ma (Fig. 9b) and negligible for 80 Ma (Fig. 9c). Because the impact of dust on climate is too small for 80 Ma, its mechanism is not analyzed.

Surface warming is obtained near the northern polar regions in all experiments (Fig. 9a-c). In 240 Ma, the climate is very warm and there is little snow and sea ice in the polar regions, so the snow-darkening effect of dust is negligible (as can be inferred from the anomalous surface shortwave radiative forcing; Fig. S7a, d). In 130 Ma, the major dust source is in the SH, it would be surprising if it has a larger snow-darkening effect in the polar region of NH than SH; the positive anomalous surface shortwave radiative forcing is more likely due to the warming effect of other processes. Rather than snow darkening, the warming of the northern polar region in both 240 Ma and 130 Ma is likely due to enhanced northward meridional heat transport (MHT) of the ocean with the MHT of atmosphere having a negative contribution (see the values new 70° *N* in Fig. S8a and 80° *N* in Fig. S8b).

**Fig. 10:**
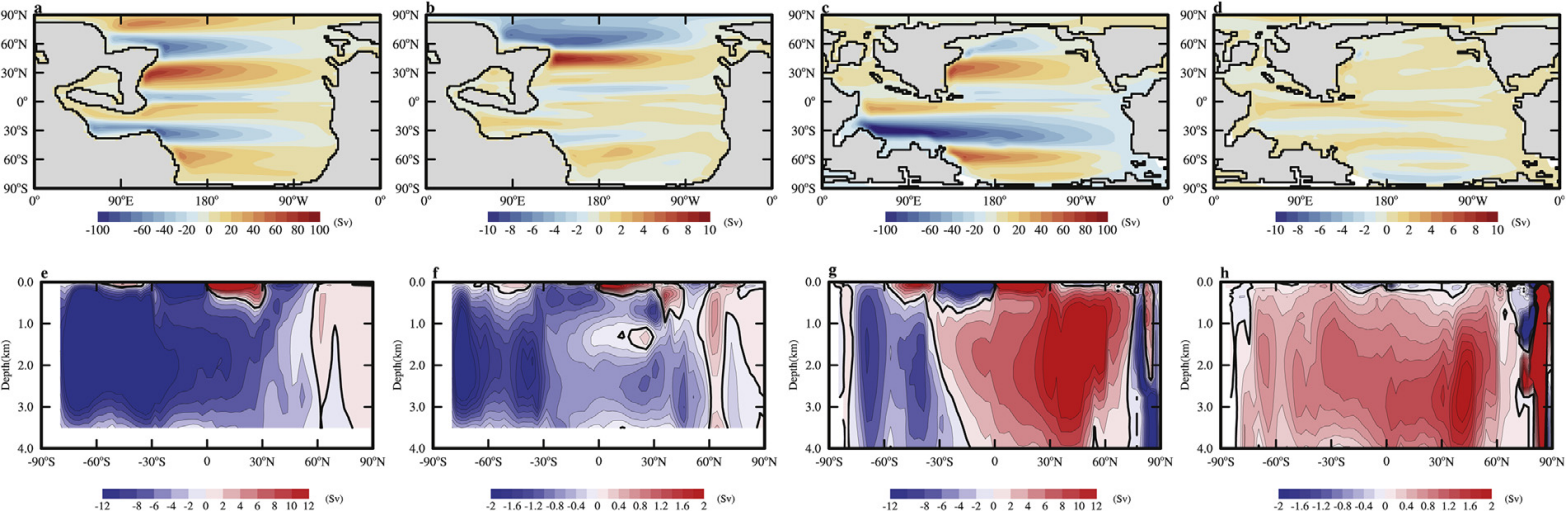
**Annual-mean (a-d) gyre (Sv) and (e-h) meridional overturning circulation (Sv) for (a, e)240Ma_7840CO_2__7840Veg and (c, g) 130Ma_2520CO_2__2520Veg, and their changes relative to (b, f) 240 Ma non-dust case and (d, h) 130 Ma non-dust case.** The gyre circulation is calculated as the barotropical streamfunction integrated from ocean bottom to surface.

Further analyses show that the enhanced northward MHT of ocean is likely dominated by the enhanced gyre (horizontal) circulation in 240 Ma while global meridional ocean circulation (MOC) in 130 Ma. The main MOC in 240 Ma is counterclockwise when the meridional section is viewed from east to west (the big blue patch in Fig. 10e); there is a weak much less extensive (in the meridonal direction) clockwise MOC in the northern polar region (the patch in light red in Fig. 10e). The counterclockwise and clockwise MOCs act to transport heat towards the south and north, respectively. The amount of heat that can be transported depends on both the strength of the MOC and the temperature difference between the upper and lower branches. Because the temperature contrast in the clockwise MOC in 240 Ma is small (both the surface temperature and bottom temperature are cold), it is unlikely to be responsible for the enhanced MHT although the strength of this MOC is enhanced when dust is present (Fig. 10f).

If not the MOC, then the gyre circulation must be responsible for the enhanced MHT of ocean in the northern polar region in 240 Ma. This is confirmed by the enhanced northern subpolar gyre circulation when dust is present (Fig. 10a, b). This stronger gyre circulation is due to the stronger mid-latitude westerly wind in the NH (Fig. S9a). This westerly wind in the NH also becomes stronger in 130 Ma (Fig. S9b) when dust is present, but the change in the northern subpolar gyre is negligible (Fig. 10c, d) probably due the presence of too much land in this region. Thus, the gyre circulation does not contribute to the warming of the northern polar region in this case. On the other hand, the major MOC in 130 Ma is clockwise (Fig. 10g) and is strengthened (Fig. 10h) when dust is present; the MHT of ocean is thus enhanced by this MOC.

The impact of dust on precipitation is small in all cases, with the maximum change obtained for 240 Ma, reaching 1.0 mm/day at a local region (Fig. 9d-f). In general, precipitation increases over land where the dust loading is heavy. This is consistent with what was found for the mid-Holocene in Zhang et al. [102]. The presence of dust absorbs sunlight and warms the middle atmosphere, which drives an upwelling and a convergence of the low-level atmosphere (not shown) as well as the water vapor.

## 4 Conclusion

In this study, the dust activity during the Mesozoic is investigated using CESM 1.2.2 combined with the vegetation model BIOME4, and the influence of continental configuration, climate and the land plant evolution on the dust cycle is quantified. To do this, three typical time periods of the Mesozoic are selected, namely 240 Ma, 130 Ma and 80 Ma, and their dust emissions and loadings are compared to those of present day. The results show that the atmospheric dust loading was 35.9, 9.7 and 3.2 Tg in these three time periods, respectively. In comparison, the dust loading of today is ∼24 Tg. It is found that the area of subtropical land area has the greatest influence on dust emission and atmospheric dust loading (Fig. 4a,b). However, because the variation of the subtropical land area is coincident with the variation of continentality (high continentality means that continents are less fragmented) for these periods, it is unclear whether continentality of continents is important. For the supercontinent in 240 Ma, a much smaller fraction of dust was deposited into the ocean with the absolute value even smaller than that of present day.

Global dust emission responds to climate change substantially, especially when the vegetation is allowed to respond to climate at the same time, but the dust loading may not change proportionally to dust emission (Table 1). For example, when *p*CO_2_ is decreased from 7840 ppmv to 280 ppmv in 240 Ma, the dust emission is increased from 1956.7 Tg/yr to 3137.9 Tg/yr but the dust loading remains almost the same. This insensitivity of dust loading to dust emission is mainly due to the concurrent change of precipitation distribution, which substantially affects the wet deposition of dust.

The evolution of land plants had little contribution to the difference in atmospheric dust loading simulated for different periods of the past 250 Myr. However, future investigations are needed since deficiency is clearly present in the simulated present-day land vegetation by the model BIOME4 (see Fig. 1). For the deep past, simply removing angiosperms and C4 plants may not be enough; the physiological parameters such as temperature thresholds could also change [103].

When only the shortwave and semi-direct effects of dust are considered as done herein, dust had small impact on the global climate during probably most part of the past 250 Myr. Locally, dust had a cooling effect that could reach 2 °C during the early Mesozoic, when the supercontinent had not broken up; the effect is negligible during other periods (Fig. 9). Warming is obtained in the northern polar region in 240 Ma and 130 Ma, which is likely due to enhanced meridional heat transport by the ocean rather than due to snow-darkening effect. The enhanced heat transport is mainly due to enhanced subpolar gyre meridional overturning circulation in 240 Ma and 130 Ma, respectively. These demonstrate rich ways of dust in influencing climate. The existence of dust generally strengthens the mid-latitude westerly winds, which help drive a stronger subpolar gyre circulation of the ocean. Dust tends to increase precipitation over land by absorbing sunlight directly and promoting upwelling of the upper atmosphere and thus convergence of lower atmosphere (and moisture).

The model deficiency in both climate and vegetation simulations and the strategy of using a globally uniform surface erodibility may induce biases in the simulated dust emission and loading. Overall, the model tends to overestimate dust emission in the subtropical region but underestimate dust emission over hotspot regions where dust emission is abnormally high. The model also tends to underestimate dust emission over regions over or surrounded by high topography such as Iranian and Tibetan Plateau of present day. The simulated dust distribution may also be biased due to the uncertainty in the reconstructed land-sea mask and continental surface topography. Nonetheless, we expect the overall pattern and the global amount of simulated dust loading simulated herein to be more or less reliable and informative.

## Declaration of competing interest

The authors declare that they have no conflicts of interest in this work.

## Appendix Supplementary materials

Image, application 1

Image, application 2
