# Supplementary material for: Simulated dust activity in typical time periods of the past 250 million years
Source: Fundam Res. 2024 Feb 13;6(2):963–73. doi: 10.1016/j.fmre.2024.02.004 (PMC13069662; doi:10.1016/j.fmre.2024.02.004)
Supplement: Supplementary file 1 [file mmc1.pdf]

Supplementary material

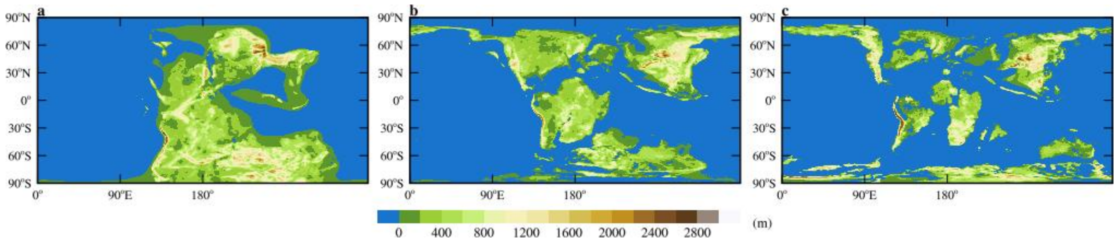

Fig. S1. The continental configuration for (a) 240Ma, (b) 130Ma, (c) 80Ma.

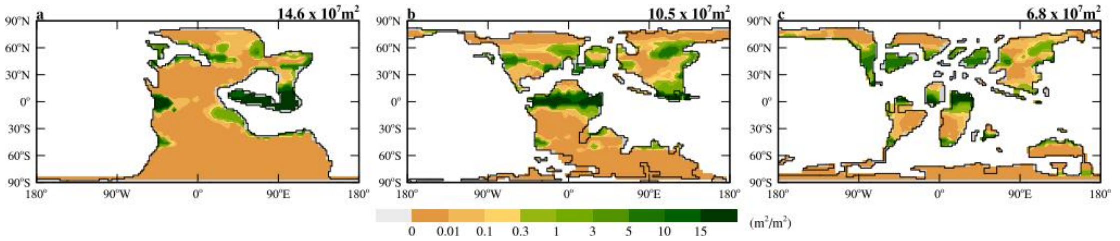

Fig. S2. The vegetation distribution obtained by CNDV/CLM4 for (a)240 Ma, (b)130 Ma, (c)80 Ma. The area of bare soil (with values <0.3) is indicated on the upper-right corner of each panel. In comparison, the area of bare soil is 2.7, 1.2, and 0.6 x10^7 m^2 for 240 Ma, 130 Ma, 80 Ma, respectively, when BIOME4 is used.

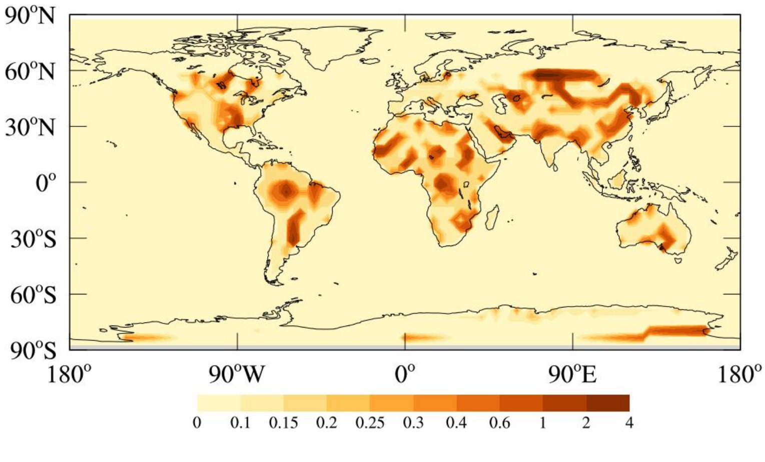

Fig. S3. Geomorphic soil erodibility.

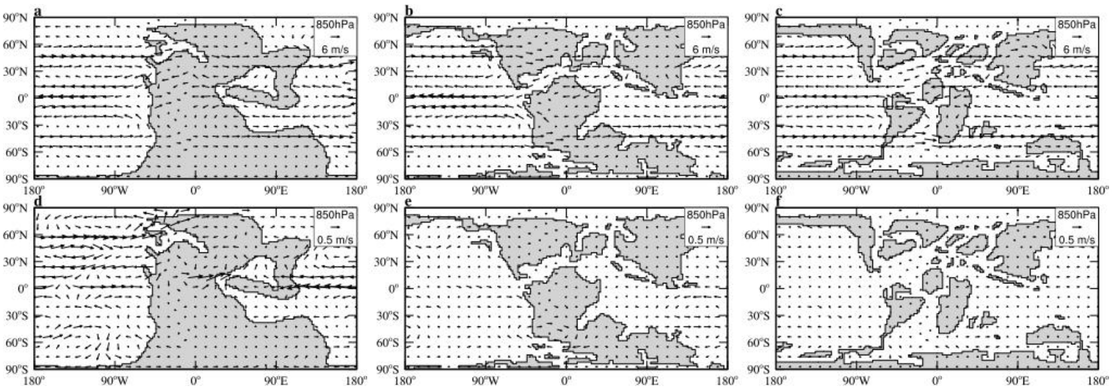

Fig. S4. Annual-mean (a-c) 850hPa winds (m/s) and (d-f) their changes relative to the non-dust cases. (a,d) 240Ma_7840CO_2_7840Veg, (b,e) 130Ma_2520CO_2_2520Veg, (c,f) 80Ma_1960CO_2_1960Veg.

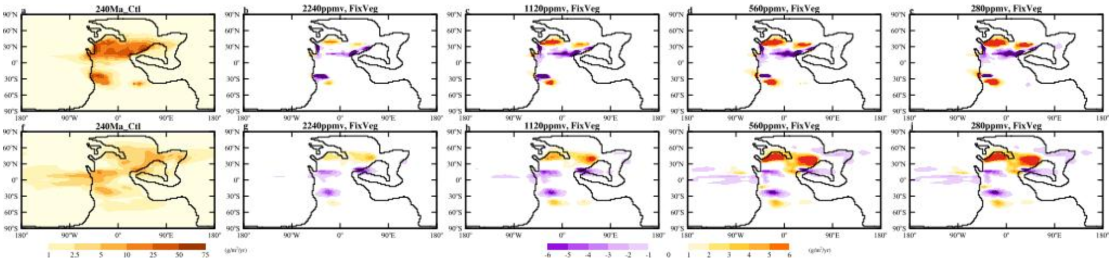

Fig. S5. Annual-mean (a-e) dry deposition (g/m^2/yr) and (f-j) wet deposition (g/m^2/yr) of dust simulated for 240 Ma under different $p$$CO_2$. The vegetation cover is fixed to that of 240Ma_7840CO_2_7840Veg. In the right four columns, the changes in respective fields relative to those of 240Ma_7840CO_2_7840Veg (left column) are shown.

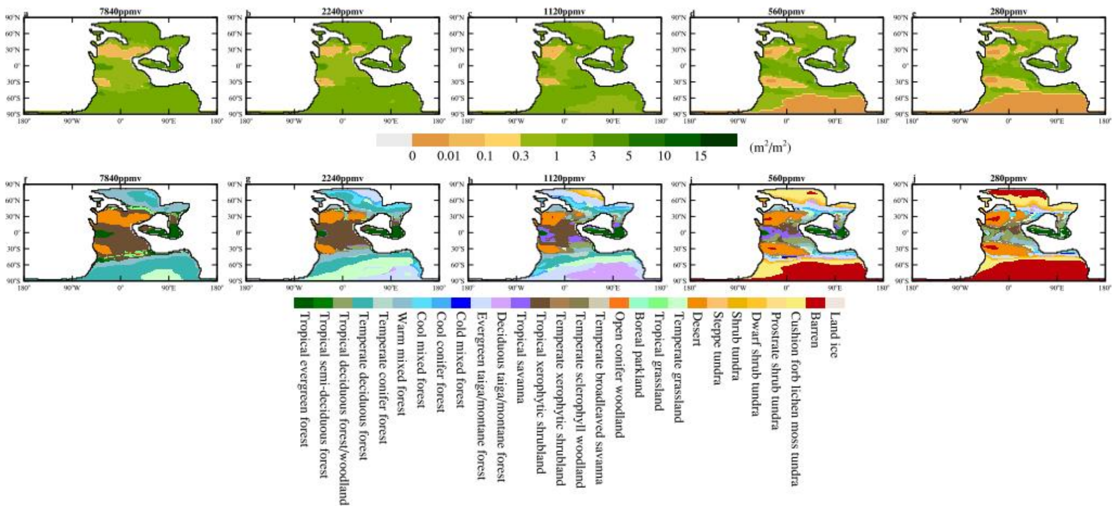

Fig. S6. (a-e) Vegetation cover (the sum of leaf plus stem area index) (m^2/m^2) and the corresponding (f-j) biomes map simulated by BIOME4 for 240 Ma under different pCO_2.

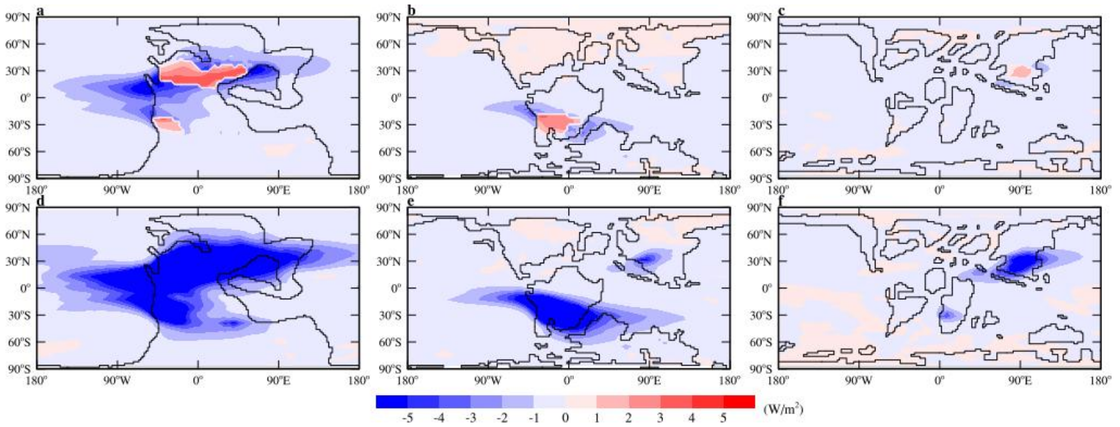

Fig. S7. Annual-mean (a-c) top-of-atmosphere and (d-f) surface shortwave radiative forcing (W/m^2) by dust. (a,d) 240Ma_7840CO_2_7840Veg, (b,e) 130Ma_2520CO_2_2520Veg, (c,f) 80Ma_1960CO_2_1960Veg. Downward is positive.

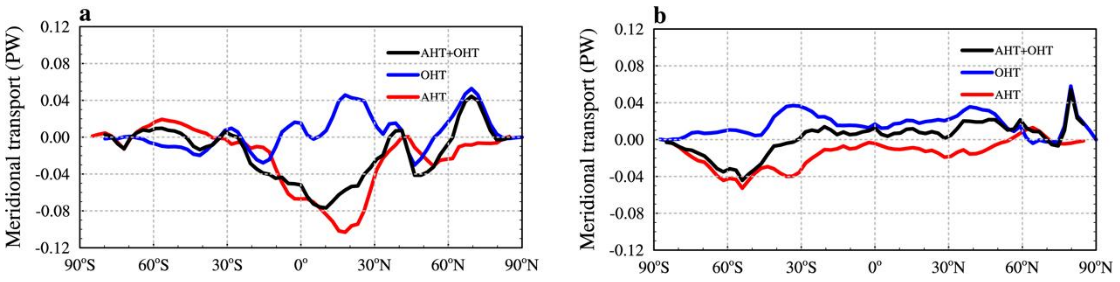

Fig. S8. Anomalous global meridional heat transport (PW) due to dust for (a) 240Ma_7840CO_2_7840Veg and (b) 130Ma_2520CO_2_2520Veg. Red, blue and black curves are the anomalous heat transport by the atmosphere, ocean, and atmosphere and ocean summed together. Northward is positive.

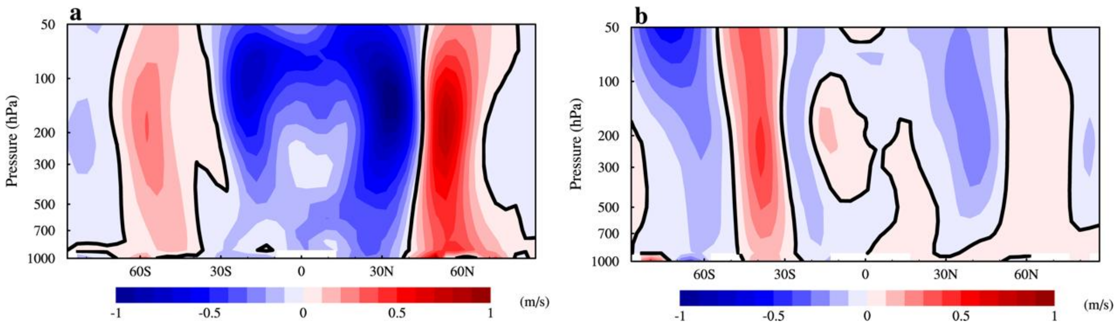

Fig. S9. Annual-mean zonal wind (m/s) due to dust for (a) 240Ma_7840CO_2_7840Veg and (b) 130Ma_2520CO_2_2520Veg.

Table S1 Dust-related geological records (aeolian sand systems and loess sites)*

Era	Location	Latitude	Longitude	Paleolatitude	Paleolongtitude	Key references
Triassic	Germanic basin	11°N-16°N	19°E-24°E	24.4°S-27.6°S	14.4°E-21.4°E	[1-3]
	French SE basin	43°N-44°N	6°E-7°E	8.7°N-9.9°N	17.9°E-18.2°E	[2, 4]
	Iberian Chain, NE Spain	40°N-42°N	0°E-4°E	4°N-7.2°N	13.7°E-17°E	[1, 2, 5]
	Holy Cross Mountains, Poland	50.7°N-51.4°N	20.5°E-21.7°E	20.1°N-21.1°N	23.3°E-23.6°E	[1, 6]
	Sudetes of NE Czech Republic	50.5°N-51.5°N	14°E-16°E	17.8°N-19.3°N	19.7°E-20.3°E	[1, 7]
	Moray Firth, Scotland	57.5°N-58.5°N	2.5°W-4°W	20.3°N-21°N	6.2°E-7.3°E	[1, 8]
	Solway basin, UK	54°N-55°N	2°W-3°W	17.1°N-17.8°N	7.9°E-8.8°E	[1, 9]
	Northern Ireland	54°N-55.5°N	6°W-8°W	16.3°N-17.5°N	4.9°E-6.5°E	[1, 10]
	Cheshire basin, UK	53.0°N-53.5°N	2.5°W-2.0°W	16°N-16.6°N	8.9°E-9°E	[1, 11]
	East Irish Sea basin, NW England	53.5°N-54.5°N	2.5°W-3.5°W	16.5°N-17.3°N	7.8°E-8.7°E	[1, 12]
	Cumbria, England	54°N-55.5°N	2°W-3.5°W	17.1°N-18.2°N	7.4°E-8.8°E	[1, 13]
	Scoresby Land, East Greenland	70°N-74°N	19°W-28°W	28.1°N-30.5°N	4.6°E-9.4°E	[1, 14]
	Central Wyoming, USA	41°N-44°N	107°W-110°W	5.9°N-10.5°N	35.1°W-39.5°W	[1, 15]
	Paradox basin, Utah, USA	37°N-39°N	109°W-111°W	3.9°N-6.4°N	41.8°W-42.4°W	[1, 16]
	Colorado and Utah, USA	37°N-41°N	102°W-114°W	0.8°N-8.5°N	32.9°W-42.9°W	[1, 17]
	Alberta, Canada	48°N-60°N	110°W-120°W	13.2°N-26.5°N	33.6°W-34.2°W	[1, 18]
	British Columbia, Canada	48°N-60°N	114°W-139°W	0-15.4°N	40.2°W-65.7°W	[1, 19]
	Fundy basin, Nova Scotia, Canada	45.5°N-46.5°N	64°W-65°W	5.3°N-6.2°N	3.7°W-4.5°W	[1, 20]
	Central High Atlas, Morocco	31°N-32°N	7°W-8°W	0.6°N-2°N	1°W-1.8°E	[1, 21]
	Argana Valley, Morocco	30.9°N-31.0°N	8.0°W-8.1°W	0.9°N-1°N	0.9°E-1°E	[1, 22]
	Auranga-Gondwana basin, Bihar, India	24°N-27°N	83°E-88°E	48°S-48.4°S	59.2°E-67.3°E	[1, 23]
	Tulong, Nyalam County, China	28.5°N	86.2°E	46.3°S	66.6°E	[24]
	Central North Sea, UK	56°N-58°N	1°W-3°E	23°N-30°N	8.7°E-16°E	[25, 26]
Early Creraceous	Neuquen basin	35°S-39°S	68°W-71°W	28°S-38°S	26°W-33°W	[27-29]
	Reconcavo-Tucano-Jatoba basins	10°S-14°S	38°W-42°W	5°S-10°S	3°W-6°W	[27, 30, 31]
	Parana basin	29°S-32°S	50°W-57°W	11°S-31°S	10°W-27°W	[27, 32]
	Sanfranciscana basin	9°S-20°S	43°W-48°W			[27, 33]
	Huab basins	20.5°S-21°S	13.75°E-14.5°E			[27, 34]
	central Namib Desert, Namibia	18°S-27°S	10°E-16°E	13.6°S-14.5°S	25.1°W-35.6°W	[35]
	Ordos basin	36°N-40°N	107°E-112°E	32.6°N-41.0°N	106°E-113°E	[36]
	Tarim basin	35°N-43°N	78°E-95°E	33.0°N-39.5°N	83°E-98°E	[36]
	Maktesh Gadol, southern Israel	30°N	35°E	30°N	6°E	[1, 37]
	Saleve Chain, southeastern France	46°N	6°E	15.6°N	36.6°E	[1, 38]
	San Marcos fault, central Coahuila, Mexico	26.75°N	102.6°W	55.9°S	24.8°E	[1, 39]
Late Cretaceous	Sanfranciscana basin	10°S-20°S	44°W-47.5°W	15.3°S-25°S	23.4°W-27.8°W	[1, 40]
	Neuquen basin	32°S-40°S	68°W-72°W	35.2°S-42.8°S	50.3°W-55.7°W	[1, 41]
	Bauru basin	18°S-25°S	47°W-54°W	23.1°S-29.5°S	27.1°W-34.9°W	[42]
	Congo basin	13°S-9°N	12°E-34°E	8.8°S-26.5°S	8.8°W-1°E	[43]
	Gobi basin	37°N-48°N	87°E-120°E	44.0°N–46.1°N	100°E-116°E	[36, 44]

	Ordos basin	36°N-40°N	107°E-112°E	32.6°N–41.0°N	83°E-98°E	[36]
	Subei basin	32°N-34°N	119°E-121°E	30.8°N–37.0°N	121°E-126°E	[36]
	Xinjiang basin	35°N-48°N	73°E-95°E	29.9°N-31.3°N	119.6°E-121.6°E	[45, 46]
	Youxian basin	39°N-41°N	114°E-115°E	27.1°N-28.6°N	118.7°E-120°E	[45, 47]
	Chuxiong basin	24°N-25°N	101°E-102°E	20.6°N-24°N	105.7°E-108.3°E	[45, 48]
	Lanping-simao basin	23°N-24°N	100°E-101°E	18.1°N-21.8°N	99.4°E-106.1°E	[45, 49]
	Kaiparowits basin, Utah, U.S.A.	37.5°N	111.6°W	45.2°N	81.9°W	[1, 50]

*It is important to note that much of the references does not give precise latitude and longitude. Many of the latitude and longitude here are given roughly based on geographic or paleogeographic maps in the references. Some of the paleolatitudes and paleolongitudes are obtained by tracing the modern latitudes and longitudes of the records back to paleo period using software GPlate. An Excel file containing this information is also provided as a supplementary file.

References

1. J. P. Rodríguez-López, L. B. Clemmensen, N. Lancaster, et al., *Archean to Recent aeolian sand systems and their sedimentary record: Current understanding and future prospects.* Sedimentology, 2014. **61**(6): p. 1487-1534.
2. S. Bourquin, M. Durand, J. B. Diez, et al., *The Permian-Triassic boundary and the early Triassic sedimentation in the western peritethys basins: An overview.* 2007. **33**(2): p. 221-236.
3. S. Bourquin, F. Guillocheau, and S. Péron, *Braided rivers within an arid alluvial plain (example from the Lower Triassic, western German Basin): recognition criteria and expression of stratigraphic cycles.* Sedimentology, 2009. **56**.
4. M. J. G. S. o. L. Durand, *The problem of the transition from the Permian to the Triassic Series in southeastern France: comparison with other Peritethyan regions.* 2006. **265**(1): p. 281-296.
5. A. Soria, C. Liesa, D. J. Rodríguez-López, et al., *An Early Triassic evolving erg system (Iberian Chain, NE Spain): Palaeoclimate implications.* Terra Nova, 2011. **23**: p. 76-84.
6. R. Gradziński, *Deep blowout depressions in the aeolian Tumlin Sandstone (Lower Triassic) of the Holy Cross Mountains, central Poland.* Sedimentary Geology, 1992. **81**(3): p. 231-242.
7. D. Uličný, *A drying-upward aeolian system of the Bohdašín Formation (Early Triassic), Sudetes of NE Czech Republic: record of seasonality and long-term palaeoclimate change.* Sedimentary Geology, 2004. **167**(1): p. 17-39.
8. A. Hurst and K. W. Glennie, *Mass-wasting of ancient aeolian dunes and sand fluidization during a period of global warming and inferred brief high precipitation: the Hopeman Sandstone (late Permian), Scotland.* 2008. **20**(4): p. 274-279.

9. M. Brookfield, *Palaeoenvironments and palaeotectonics of the arid to hyperarid intracontinental latest Permian- late Triassic Solway basin (U.K.).* Sedimentary Geology, 2008. **210**: p. 27-47.
10. J. Buckman, P. S. Doughty, M. Benton, et al., *Palaeoenvironmental interpretation of the Triassic Sandstone of Scrabo, County down, Northern Ireland: Ichnological and sedimentological studies indicating a mixed fluviatile-aeolian succession.* Irish Journal of Earth Sciences, 1997. **16**: p. 85-102.
11. J. P. Bloomfield, M. F. Moreau, and A. J. Newell, *Characterization of permeability distributions in six lithofacies from the Helsby and Wilmslow sandstone formations of the Cheshire Basin, UK.* 2006. **263**(1): p. 83-101.
12. N. Meadows, *The correlation and sequence architecture of the Ormskirk Sandstone Formation in the Triassic Sherwood Sandstone Group of the East Irish Sea Basin, NW England.* Geological Journal, 2006. **41**: p. 93-122.
13. R. P. Barnes, K. Ambrose, D. W. Holliday, et al., *Lithostratigraphical subdivision of the Triassic Sherwood Sandstone Group in west Cumbria.* 1994. **50**(1): p. 51-60.
14. L. B. Clemmensen, *Alternating aeolian, sabkha and shallow-lake deposits from the Middle Triassic Gipsdalen Formation, Scoresby Land, East Greenland.* Palaeogeography, Palaeoclimatology, Palaeoecology, 1978. **24**(2): p. 111-135.
15. A. P. Irmen and C. F. Vondra, *Aeolian sediments in lower to middle (?) Triassic rocks of central Wyoming.* Sedimentary Geology, 2000. **132**(1): p. 69-88.
16. T. Lawton and B. Buck, *Implications of diapir-derived detritus and gypsic paleosols in Lower Triassic strata near the Castle Valley salt wall, Paradox Basin, Utah.* Geology, 2006. **34**: p. 885–888.
17. R. F. Dubiel. *Sedimentology and depositional history of the Upper Triassic Chinle Formation in the Uinta, Piceance, and Eagle basins, northwestern Colorado and northeastern Utah*. 1992.
18. G. R. Davies, T. F. Moslow, and M. D. J. B. o. C. P. G. Sherwin, *The Lower Triassic Montney Formation, West-Central Alberta.* 1997. **45**: p. 474-505.
19. B. R. Nassichuk. *Sedimentology, diagenesis and reservoir development of the lower triassic montney formation, northeastern British Columbia*. 2000.
20. S. Leleu and A. Hartley, *Controls on the stratigraphic development of the Triassic Fundy Basin, Nova Scotia: Implications for the tectonostratigraphic evolution of Triassic Atlantic rift basins.* Journal of The Geological Society - J GEOL SOC, 2010. **167**: p. 437-454.
21. I. Fabuel-Perez, J. Redfern, and D. Hodgetts, *Sedimentology of an intra-montane rift-controlled fluvial dominated succession: The Upper Triassic Oukaimeden Sandstone Formation, Central High Atlas, Morocco.* Sedimentary Geology - SEDIMENT GEOL, 2009. **218**: p. 103-140.
22. N. Mader and J. Redfern, *A sedimentological model for the continental Upper Triassic Tadrart Ouadou Sandstone Member: recording an interplay of climate and tectonics (Argana Valley; South-west Morocco).* Sedimentology, 2011. **58**: p. 1247-1282.
23. D. P. Sen and T. C. Sinha, *Triassic aeolian sedimentation in the Auranga Gondwana Basin, Bihar, India.* Sedimentary Geology, 1985. **43**(1): p. 277-300.
24. X. Jiang, T. X. Zhu, X. T. Feng, et al., *Discovery of the Late Triassic coastal aeolian dune in South Tibet Tethys and its significance.* 2003. **30**: p. 447-452.

25. A. D. Wilkins, A. Hurst, M. J. Wilson, et al., *Palaeo-environment in an ancient low-latitude, arid lacustrine basin with loessite: The Smith Bank Formation (Early Triassic) in the Central North Sea, UK Continental Shelf.* Sedimentology, 2018. **65**(2): p. 335-359.
26. M. J. Wilson, A. Hurst, A. D. Wilkins, et al., *Mineralogical evidence for multiple dust sources in an early Triassic loessite.* Sedimentology, 2019. **67**(1): p. 239-260.
27. C. M. S. Scherer, R. G. Mello, J. P. F. Ferronatto, et al., *Changes in prevailing surface-palaeowinds of western Gondwana during Early Cretaceous.* Cretaceous Research, 2020. **116**.
28. A. Argüello Scotti and G. D. Veiga, *Sedimentary architecture of an ancient linear megadune (Barremian, Neuquén Basin): Insights into the long-term development and evolution of aeolian linear bedforms.* 2019. **66**(6): p. 2191-2213.
29. G. D. Veiga, L. A. Spalletti, and S. Flint, *Aeolian/fluvial interactions and high-resolution sequence stratigraphy of a non-marine lowstand wedge: the Avilé Member of the Agrio Formation (Lower Cretaceous), central Neuquén Basin, Argentina.* 2002. **49**(5): p. 1001-1019.
30. J. P. Formolo Ferronatto, C. M. d. S. Scherer, E. G. de Souza, et al., *Genetic units and facies architecture of a Lower Cretaceous fluvial-aeolian succession, São Sebastião Formation, Jatobá Basin, Brazil.* Journal of South American Earth Sciences, 2019. **89**: p. 158-172.
31. F. Wiederkehr. *Análise tectono-estratigráfica das formações itaparica e água grande (Bacia do Recôncavo, Bahia)*. 2010.
32. G. Bertolini, J. C. Marques, A. J. Hartley, et al., *Controls on Early Cretaceous desert sediment provenance in south-west Gondwana, Botucatu Formation (Brazil and Uruguay).* Sedimentology, 2020. **67**(5): p. 2672-2690.
33. P. C. Mescolotti, F. G. Varejão, L. V. Warren, et al., *The sedimentary record of wet and dry eolian systems in the Cretaceous of Southeast Brazil: stratigraphic and paleogeographic significance.* 2019. **49**.
34. Mountney and Howell, *Aeolian architecture, bedform climbing and preservation space in the Cretaceous Etjo Formation, NW Namibia.* 2000. **47**(4): p. 825-849.
35. A. S. Goudie and F. J. G. A. S. A. Eckardt, Physical Geography, *The evolution of the morphological framework of the central Namib Desert, Namibia, since the early Cretaceous.* 1999. **81**(3): p. 443-458.
36. H. Hasegawa, R. Tada, X. Jiang, et al., *Drastic shrinking of the Hadley circulation during the mid-Cretaceous Supergreenhouse.* Climate of the Past, 2012. **8**(4): p. 1323-1337.
37. E. Azmon, *Ventispheres: granule-pebble spheres of eolian origin in lower cretaceous and recent rocks of Israel.* Sedimentary Geology, 1982. **33**(1): p. 57-71.
38. P. Kindler and E. Davaud, *Recognizing eolianites in thin section: review and case study*. 2001. p. 141-150.
39. G. González-Naranjo, R. Molina Garza, and G. Chávez-Cabello, *Paleomagnetic study of Jurassic and Cretaceous rocks north of San Marcos fault, central Coahuila, México.* Geofísica Internacional, 2007. **47**.
40. A. L. D. Spigolon and C. J. S. d. J. B. J. o. G. Alvarenga, *Fácies e elementos arquiteturais resultantes de mudanças climáticas em um ambiente desértico: Grupo Urucuia (Neocretáceo), Bacia Sanfranciscana.* 2002. **32**: p. 579-586.
41. P. Armas, M. C. M. Garrido, and M. L. Sánchez. *Areniscas eólicas y mareales del Cretácico Superior de la Cuenca Neuquina (Argentina). Análisis petrográfico y de procedencia*. 2012.

42. L. A. Fernandes, A. B. de Castro, and G. Basilici, *Seismites in continental sand sea deposits of the Late Cretaceous Caiuá Desert, Bauru Basin, Brazil.* Sedimentary Geology, 2007. **199**(1-2): p. 51-64.
43. P. Giresse, *Mesozoic–Cenozoic history of the Congo Basin.* Journal of African Earth Sciences, 2005. **43**(1-3): p. 301-315.
44. H. Hasegawa, R. Tada, N. Ichinnorov, et al., *Lithostratigraphy and depositional environments of the Upper Cretaceous Djadokhta Formation, Ulan Nuur basin, southern Mongolia, and its paleoclimatic implication.* Journal of Asian Earth Sciences, 2009. **35**(1): p. 13-26.
45. J. P. Rodríguez-López and C. Wu, *Recurrent deformations of aeolian desert dunes in the cretaceous of the South China Block: Trigger mechanisms variability and implications for aeolian reservoirs.* Marine and Petroleum Geology, 2020. **119**.
46. C. Wu, J. P. Rodríguez-López, C. Liu, et al., *Late Cretaceous climbing erg systems in the western Xinjiang Basin: Palaeoatmosphere dynamics and East Asia margin tectonic forcing on desert expansion and preservation.* Marine and Petroleum Geology, 2018. **93**: p. 539-552.
47. T. Ge, J. Liu, and L. Fan. *Magnetostratigraphy of the Red Beds in Hengyang Basin*. in *Chinese Science Abstracts Series B*. 1995.
48. D. Xi, X. Wan, G. Li, et al., *Cretaceous integrative stratigraphy and timescale of China.* 2019. **62**: p. 256-286.
49. G. Li, C. Wu, J. P. Rodríguez-López, et al., *Mid-Cretaceous aeolian desert systems in the Yunlong area of the Lanping Basin, China: Implications for palaeoatmosphere dynamics and paleoclimatic change in East Asia.* Sedimentary Geology, 2018. **364**: p. 121-140.
50. E. L. Simpson, H. L. Hilbert-Wolf, W. S. Simpson, et al., *The interaction of aeolian and fluvial processes during deposition of the Upper Cretaceous capping sandstone member, Wahweap Formation, Kaiparowits Basin, Utah, U.S.A.* Palaeogeography, Palaeoclimatology, Palaeoecology, 2008. **270**(1): p. 19-28.
